# Photoluminescent Nanocellulosic Film for Selective Hg^2+^ Ion Detection

**DOI:** 10.3390/polym16111583

**Published:** 2024-06-03

**Authors:** Jing Sun, Wenwen Fang, Afroza Akter Liza, Rui Gao, Junlong Song, Jiaqi Guo, Orlando J. Rojas

**Affiliations:** 1 Jiangsu Co-Innovation Center for Efficient Processing and Utilization of Forest Resources and International Innovation Center for Forest Chemicals and Materials, Nanjing Forestry University, Nanjing 210037, China; 18816213069@163.com (J.S.); lizakhulna@gmail.com (A.A.L.); gaorui2127@163.com (R.G.); junlong.song@njfu.edu.cn (J.S.); 2 Department of Bioproducts and Biosystems, Aalto University, P.O. Box 16300, 00076 Helsinki, Finland; wenwen.fang@aalto.fi; 3 Bioproducts Institute, Department of Chemical and Biological Engineering, Department of Chemistry and Department of Wood Science, University of British Columbia, 2360, East Mall, Vancouver, BC V6T 1Z3, Canada

**Keywords:** cellulose nanofiber, luminescent nanocomposite, anti-counterfeiting, heavy metal detection

## Abstract

We developed a highly sensitive solid-state sensor for mercury detection by stabilizing red-sub-nanometric fluorescent gold nanoclusters (AuNC, 0.9 ± 0.1 nm diameter) with bovine serum albumin in a matrix composed of cellulose nanofibrils (CNF) (BSA-AuNC/CNF). The main morphological and optical features of the system were investigated via atomic force/transmission electron microscopy and UV-Vis/fluorescence spectroscopy. The hybrid film (off-white and highly transparent) showed strong photoluminescene under UV irradiation. The latter is assigned to the AuNC, which also increase the ductility of the emitting film, which was demonstrated for high sensitivity Hg^2+^ detection. When used as a sensor system, following AuNC printing on CNF hybrid films, a limit of detection <10 nM was confirmed. What is more, nanocellulose films have a high pore structure and selective separation properties, showcasing a wide range of potential applications in many fields such as water treatment and oil–water separation.

## 1. Introduction

There has been an increased demand for advanced materials derived from bio-based renewable resources [1,2], especially if assisted by cost-effective biopolymers such as cellulose, hemicellulose, lignin, and starch [3]. These biopolymers act as crucial building blocks for creating functional materials that are economically feasible and sustainably viable [4]. Plant-based cellulose has the potential to serve as a scaffold for functional ligands, promoting the development of functional paper-based sensors and actuators. As a new type of nanomaterial, cellulose nanofibers (CNF) play an important role in the field of polymer science. Additionally, CNF have garnered significant interest owing to their distinctive characteristics [5]. Unlike native cellulose or microcrystalline cellulose, CNF stands out for its specific surface area, crystallinity, biocompatibility, biodegradability, and high Young’s modulus, enabling the preparation of polymer materials with specialized functions. For example, CNF can be used to prepare conductive polymers, antibacterial polymers, self-healing polymers, etc. These materials have a wide range of application prospects in electronics, medicine, environmental protection, and other fields [6,7]. CNF typically consist of nanoscale cellulose fibrils characterized by a narrow width (ranging from 5–20 nm) and a length in the micron scale [8]. Recently, CNF-based luminescent nanocomposites have been adopted as substitutes for fossil carbon platforms used for detection and bioimaging [9]. In this regard, luminescence has gained traction for the detection of metal ions, sensing, and bioimaging [10]. Most reported techniques rely on fluorescent substances deposited on paper, which affect the underlying structure of the support [11]. However, traditional cellulose fibers are typically too coarse. This makes it difficult to effectively stabilize nanosized fluorescent nanoparticles [12]. Consequently, current cellulose-based sensors suffer from inadequate stability, recovery efficiency, and longevity. To address these challenges, researchers have explored CNF-based luminescent nanocomposites in the form of 3D membranes [13], offering a substantial surface area for efficient immobilization and intense fluorescence [14]. Most relevant to the area is affording highly sensitive identification systems for toxic ions, such as mercury (Hg^2+^), which are associated with harmful effects on ecosystems and organisms [15]. Hg^2+^ is predominantly emitted from industrial processes, including chemical manufacturing, the burning of fossil fuels, and the incineration of solid waste [16]. Hg^2+^ is readily soluble in water, persistent, and widely distributed [17,18].

Mercury has the potential to accumulate in various human organs and tissues. It binds with sulfur-rich proteins and enzymes, causing organ dysfunction and devastating effects on the entire central nervous system of humans. Remarkably, even at extremely low concentrations, Hg^2+^ is capable of causing severe health impairments.

Despite the negative impact of metal ions on proteins, there is an opportunity to repurpose this interaction for sensing applications, as demonstrated with Hg^2+^ [19]. Traditionally, the detection of Hg^2+^ ions has been accomplished through the utilization of chromatography or cold vapor generation [20] followed by analysis using inductively connected plasma mass spectrometry [21], ion selective electrodes [22], atomic absorption spectrometry [23], and atomic fluorescence spectroscopy [24]. The majority of these methods rely on the formation of complexes between mercury ions and single-channel detection, which have an impact on both selectivity and sensitivity [25,26].

Herein, we consider the detection of Hg^2+^ by following changes in the luminescence response of AuNC in an aqueous matrix, which involves the fluorescence quenching generated by the metal ion with an “on-off” signal [27]. Current efforts have focused on the examination of one or more stabilizers or protective molecules, e.g., to prevent AuNC self-aggregation [28]. The authors of previous studies have successfully synthesized BSA-AuNC with notable fluorescence properties, including peak emission at 640 nm and a fluorescence quantum efficiency of approximately 6%. In addition, it has been shown that the fluorescence of BSA-AuNC is suppressed by the presence of Hg^2+^, leading to successful screening for Hg^2+^ [27].

In this work, we developed a simple but highly stable AuNC/CNF solid-state sensing film for the detection of Hg^2+^ based on its interactions with Au^+^. Red fluorescent AuNC embedded in a transparent CNF matrix enhanced both fluorescence intensity and film durability. As a result, unprecedented sensitivity and stability were obtained, offering a cutting-edge alternative for efficient and rapid Hg^2+^ detection and the development of advanced security features.

## 2. Materials and Methods

### 2.1. Materials

Tetrachloroauric (III) acid trihydrate (HAuCl_4_·3H_2_O, 99%) and bovine serum albumin (BSA, lyophilized powder, ≥98%) were purchased from Sigma-Aldrich, St. Louis, MI, USA. All chemicals and materials in this work were of analytical grade and used with no further purification. Milli-Q water (>18 MΩ·cm) was used in all experiments.

Cellulose nanofibrils (CNF) were prepared from never-dried, fully bleached, and fine-free (SCAN-M 6:69) sulfite hardwood (birch) fibers (Finnish pulp mill, kappa number = 1 and DP = 4700) [29]. The fibers were washed and converted to the sodium form to control both the counter-ion type and ionic strength. The washed fibers were disintegrated through a high-pressure fluidizer (Microfluidics M110P, Microfluidics Int. Co., Newton, MA, USA) using 6 passes. No chemical or enzymatic pre-treatment was used prior to disintegration.

### 2.2. Synthesis of Luminescent BSA-Protected Gold Nanocluster (BSA-AuNC)

BSA-AuNC was synthesized following the procedure previously described [30]. Briefly, 50 mL HAuCl_4_·3H_2_O solution (*c* = 1 mM) was added to BSA solution (5 mL, 50 mg/mL, 37 °C) and stirred vigorously. Then, 5 mL trisodium citrate (*c* = 1 wt%) was added rapidly when the solution boiled. While the red-wine-colored solution formed, the colloidal solution was refluxed for 30 min in order to ensure complete reduction. After cooling to room temperature, the formed colloidal particles (BSA-AuNC) were dialyzed (MWCO = 1000) against distilled water for 7 days.

### 2.3. AuNC/CNF Nanocomposites

AuNC was supported in a CNF matrix by following our previously reported protocol [31]. In short, a diluted CNF dispersion (*c* = 0.25 wt%) was added to the BSA-AuNC system while stirring, leading to a homogeneous colloidal mixture. The well-mixed dispersion was concentrated via rotary evaporation and evaporated in a Petri dish at room temperature.

### 2.4. Imaging

Atomic force microscopy (AFM) was conducted using a Vortis scanning probe microscope with a Nanoscope V controller operated in tapping mode (Bruker Corparation, Billerica, MA, USA) (silicon nitride tip; nominal end radius: 1 nm; spring constant: 0.25 N/m; resonance frequency: 110 kHz). The CNF was diluted to the concentration of ca. 0.05 wt% and deposited onto a fresh silica wafer.

The shape and distribution of BSA-AuNC were studied by using a high-resolution transmission electron microscope (HRTEM, Tecnai F20 G2, Eindhoven, The Netherlands) operated at 200 kV accelerating voltage. The size distribution was analyzed using the TEM images through the use of ImageJ software (v1.8.0.345, National Institutes of Health, Bethesda, MD, USA). The fracture surface of the CNF/AuNCs nanocomposites was imaged using a Zeiss SEM (Zeiss, Jena, Germany) after sputter-coating an ultrathin carbon layer. The operation voltage of the SEM was set at 1.5 kV to avoid damage to the samples. Finally, the crystal structure was investigated via an electron diffraction ring pattern.

### 2.5. Spectroscopic Analyses

The absorption and transmittance spectra of BAS-AuNC and its nanocomposites were determined with a UV–vis Spectrophotometer UV-2600 (Shimadzu, Kyōto-shi, Japan). The wavelength in the range of 300–1000 nm was recorded. The luminescence properties were investigated using a fluorescence spectrometer (PerkinElmer, Waltham, MA, USA). The 1D and 2D fluorescence spectra of AuNC solution and AuNC/CNF nanocomposites were collected.

### 2.6. Mechanical Strength and Printing

The mechanical properties of the prepared AuNC/CNF nanocomposites were determined using a DEBEN mini tester equipped with a 200 N load cell. Prior to measurements, the specimens were conditioned overnight at 23 °C and 50% relative humidity (RH). The specimens had a size of 2 mm × 15 mm, and at least 6 samples were measured at the strain rate of 1 mm/min. Finally, inkjet printing was conducted using a Fuji film Dimatix Materials Printer (DMP-2831, Santa Clara, CA, USA) equipped with shear mode DOD print heads. The cartridge module was Dimatix DMC-11610 (nominal drop volume ≈ 10 pL).

## 3. Results and Discussion

### 3.1. BSA-Protected Gold Nanoclusters (BSA-AuNCs) and Cellulose Nanofibrils (CNF)

**Figure 1:**
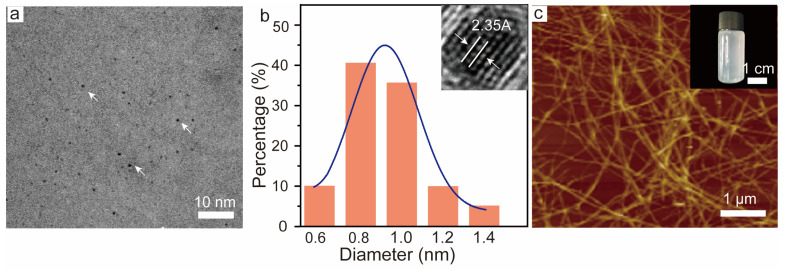
AuNC and CNF morphology. (**a**) Transmission electronic microscope (TEM) image of AuNC, the arrows point to AuNC; (**b**) size distribution of AuNC and high-resolution TEM image of an individual AuNC (inset); (**c**) atomic force microscope (AFM) image of the surface topography of CNFs and a digital photo of 0.5 wt% CNF dispersion (inset).

A starting point of the engineering of the photoluminescent nanocomposite film based on CNF is the synthesis of a waterborne luminescent component that is compatible and easily integrated into the fibril network. For this purpose, the water-based gold nanoclusters (AuNC) system is highly suitable, particularly when stabilized using bovine serum albumin (BSA), which also acts as a reducing agent for AuNC. The synthesized BSA-AuNC showed a spherical morphology and uniform size in HR-TEM (Figure 1a). The TEM analysis revealed a size of 0.91 ± 0.1 nm based on the frequency distribution histogram, with a lattice spacing of 2.35 Å, corresponding to the (1 0 1) lattice planes of Au [32]. The optical transparency of the composite film was significantly influenced by the morphology of the CNF (see the CNF suspension prepared using the microfluidizer from bleached sulfite hardwood fibers, inset of Figure 1c). The atomic force microscope (AFM) image showed a consistent size distribution with a diameter ranging from 10–20 nm and an overall length of several μm. The width of the CNF was much smaller than the wavelength of visible light (380–780 nm), as illustrated in Figure 1c. This indicates that the CNF matrix exhibits high efficiency in transmitting light. This unique property not only enhances optical transparency but also contributes to a high photoluminescent intensity within the composite film.

### 3.2. BSA-AuNC Photoluminescence

**Figure 2:**
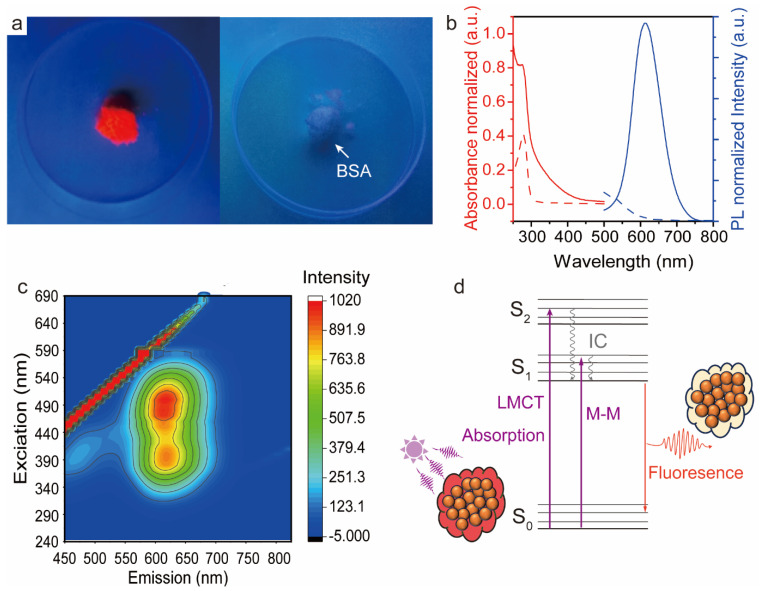
Photoluminescence of AuNC. (**a**) Digital picture of freeze-dried AuNC powder and purified BSA powder under UV irradiation (*λ* = 365 nm); (**b**) absorption and emission spectra of photoluminescent BSA-AuNC (BSA powder is shown with a dashed line and BSA-AuNC is shown with a solid line). (**c**) Three-dimensional (3D) luminescence spectra of BSA-AuNC and (**d**) schematic diagram explaining the photoluminescent mechanism of BSA-AuNC.

High red fluorescence s detected when a freeze-dried AuNC powder was excited with UV light (λ = 365 nm) (Figure 2a, left). In contrast, the freeze-dried BSA powder appeared white and did not exhibit any emission under UV light (Figure 2a, right). The fluorescent AuNC dispersion (*c* = 1 wt%) presented excitation and emission peaks at 280 and 640 nm, respectively (Figure 2b). The BSA solution (*c* = 1 wt%) presented a subtle fluorescence emission (*λ*_em_ = 480 nm) under excitation at 280 nm, attributed to the aromatic side groups in the amino acid residues (tryptophan, tyrosine, and phenylalanine) [33]. Further studies were conducted on the BSA-AuNC system using a fluorescent spectrometer and three-dimensional (3D) fluorescence spectra were created (Figure 2c). To our surprise, BSA-AuNC displayed two excitation peak wavelengths, and the emission intensity depended on the excitation wavelength. Notably, emissions were significantly stronger at 490 nm excitation compared to 390 nm. Referencing the Jablonski diagram, fluorescence is the spontaneous emission of light during the transition of the system from its lowest vibrational energy level to an excited singlet state S_1_ and back to the ground state S_0_ [34]. Based on this, we speculate that the ultraviolet absorbance of BSA-AuNC arises from transitions between molecular orbitals featuring high ligand contribution to orbitals with a strong metal character. This phenomenon is known as ligand-to-metal charge transfer (LMCT), ultimately leading to the red emission of BSA-AuNC from the charge-transfer excited state [35]. In summary, we successfully synthesized highly luminescent AuNC with red emission.

### 3.3. Photoluminescent BSA-AuNC/CNF Hybrid Film

**Figure 3:**
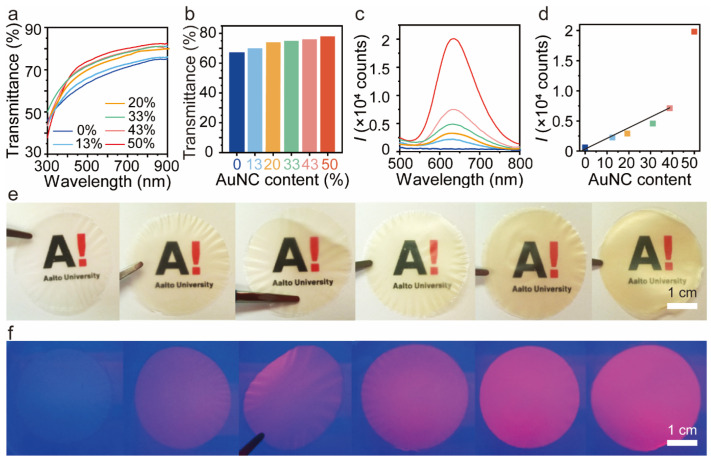
Optical properties of the BSA-AuNC/CNF films. (**a**) Transmittance of the BSA-AuNC/CNF film as a function of BSA-AuNC loading. (**b**) Histogram profile of the transparency of BSA-AuNC/CNF films at λ = 600 nm. (**c**,**d**) Emission spectra and luminescence intensity at 640 nm of the BSA-AuNC/CNF films as a function of BSA-AuNC loading. Photographs of transparent BSA-AuNC/CNF films at given BSA-AuNC loading (increased loading from left to right: 0, 13, 20, 33, 43, and 50%) under natural (**e**) and UV (**f**) light. Note: Color codes are used to represent the different BSA-AuNC concentrations. The intensity map with varied BSA-AuNC content in (**d**) corresponds to the concentrations shown in (**a**,**b**).

The general strategy for the preparation of BSA-AuNC/CNF films started with the formation of the nanostructure by incorporating BSA-AuNC into the CNF through a water-borne casting route [36]. During this process, BSA-AuNC was adsorbed onto the CNF. To design the nanocomposite, we adjusted the pH close to the BSA isoelectric point to ensure good mixing with anionic CNF, preventing aggregation in water. When mixed, BSA-AuNC attached to the surface of the CNF, and evaporation of the water phase using given BSA-AuNC/CNF ratios produced transparent and homogeneous hybrid films (ca. 30 μm thickness). The transparency of the neat CNF film (AuNC = 0 wt%) reached nearly 70%. This transparency increased, slightly above 70%, with the increased AuNC content (Figure 3a,b). This increase is attributed to the reduction in the diffuse reflection of light while the nanopores of the CNF were filled with BSA-AuNCs [37]. We investigated the luminescence of BSA-AuNC/CNF nanocomposite film with various BSA-AuNC/CNF ratios. The emission peak presented no obvious displacement under varying AuNC content and remained at 640 nm. As expected, the fluorescence intensity showed a gradual increase with the increased concentration of BSA-AuNC (Figure 3c). Due to the unique quantum dot properties of AuNC and their enhanced stability when modified with BSA, the interaction between them may be strengthened when more BSA-AuNC are loaded onto the CNF membrane, leading to an increase in luminous intensity [38]. Despite the difference in AuNC content, the consistent emission peak at 640 nm without significant displacement is due to the fact that the size of AuNC is usually controlled within a few nanometers. In this size range, the emission spectrum of a nanoparticle is often determined by its electronic structure, not just its concentration [39]. Therefore, even if the concentration of AuNC (AuNC content) changes, the peak of their emission spectrum (such as 640 nm) is likely to remain consistent as long as their size remains the same. A plot of fluorescence intensity and BSA-AuNC/CNF ratio showed a linear relationship up to 75 wt% of AuNC (43 wt%). The neat CNF and BSA-AuNC/CNF films showed high transparency and off-white color, respectively (Figure 3e). The digital photos of BSA-AuNC/CNF films with different BSA-AuNC content under ultraviolet light (*λ* = 365 nm) showed a similar tendency (fluoresce spectra, Figure 3f).

### 3.4. Mechanical Strength of BSA-AuNC/CNF Films

**Figure 4:**
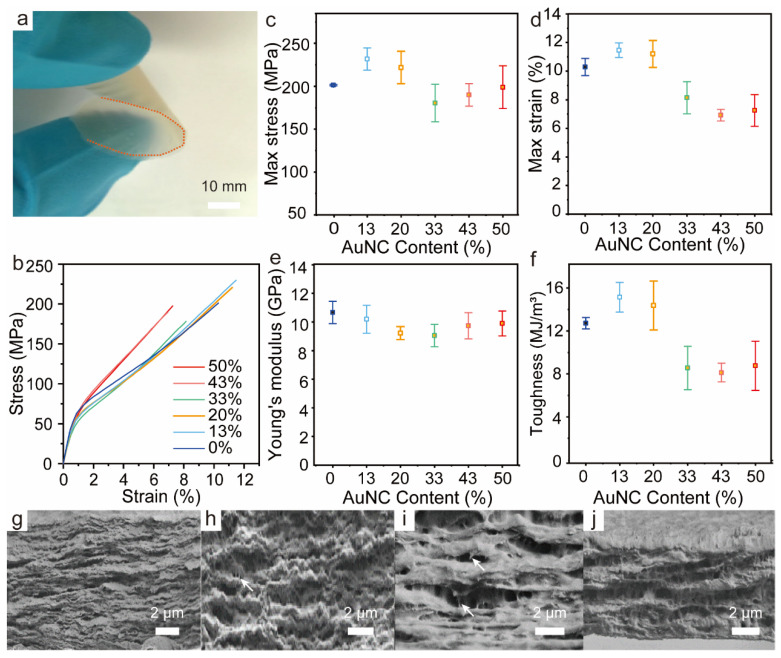
Mechanical properties of BSA-AuNC/CNF films. (**a**) Digital photo showing the flexibility of a hybrid film. (**b**) Representative stress–strain curves of BSA-AuNC/CNF films. (**c**) Tensile strength (*σ*_b_), (**d**) strain-to-failure (*ε*_b_), (**e**) Young’s modulus (*E*), and (**f**) toughness (*U*_t_) of BSA-AuNC/CNF films with given BSA-AuNC loading. (**g**–**j**) Cross-sectional SEM images of fractured BSA-AuNC/CNF films loaded with given BSA-AuNC loading. CNF set as 100 with varied AuNC amount in. (arrows in (**h**,**i**) represent ductile fiber from the slippage of the fibrillar network).

We conducted an investigation into how the loading of BSA-AuNC/CNF influences the mechanical properties of films under conditions of 23 °C and 50% relative humidity (RH). From a macroscopic viewpoint, BSA-AuNC/CNF films were flexible up to an AuNC/CNF loading of 33 wt% (Figure 4a). Figure 4b illustrates the tensile curves of BSA-AuNC/CNF films as a function of AuNC/CNF content (50% RH). The incorporation of AuNC/CNF resulted in the BSA-AuNC/CNF films exhibiting a slightly more ductile nature, evidenced by a decrease in Young’s modulus (E) and tensile strength (σb), along with slight increases in strain-to-failure (εb) and toughness (U_t_). Starting from the neat CNF film and by adding 20 wt% BSA-AuNC, *ε*_b_, *σ*_b,_ and *U*_t_ increased from 10.1%, 200 MPa, and 12.5 MJ/m^3^ to 11.07%, 220 MPa and 14.4 MJ/m^3^, respectively. Meanwhile, *E* was reduced from 10.6 to 9.0 GPa. The smooth transition of the films, from a state of stiffness and strength to one of ductility, is attributed to the presence of BSA, the soft phase, which allows dissipative CNF frictional sliding [40]. It is worth noting that excess BSA disrupts the hydrogen bonding that originally existed between cellulose nanofibrils [41]. Figure 4g–j show the cross-sectional SEM images of the corresponding fracture surfaces of CNF and BSA-AuNC/CNF films to elucidate the different deformation behaviors. The neat CNF film showed a featureless fracture surface (Figure 4g), reminiscent of the untoughened nanocomposite structure. In contrast, the inclusion of BSA-AuNC in the CNF network leads to a strong pull-out of mesoscale layers (Figure 4h,i), which stems from a ductile behavior given the effect of slippage of the fibrillar network [42]. More interestingly, there was no obvious BSA-AuNC aggregation. The elongation performance was comparable to some plastics, such as polylactic acid [43]. This noteworthy enhancement in mechanical properties, coupled with the fluorescence response, establishes strong groundwork for diverse applications including smart packaging materials and anti-counterfeiting measures.

### 3.5. Hg^2+^ Detection with Printed BSA-AuNC onto CNF Film

**Figure 5:**
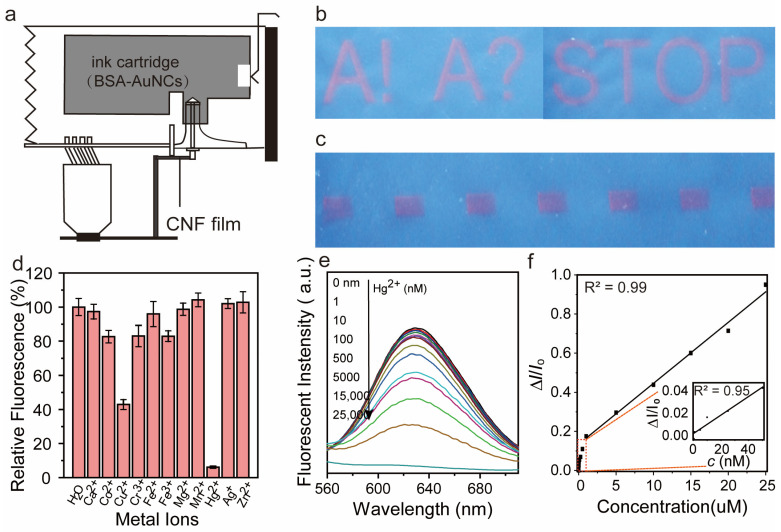
Sensitivity and selectivity of BSA-AuNC/CNF film obtained through printing. (**a**) BSA-AuNC dispersion was used as fluorescent ink in a Fujifilm inkjet printer; (**b**,**c**) a defined, printed pattern showing the luminescence under UV irradiation. (**d**) Relative luminescence intensity after exposure to various heavy metal ions. Luminescence spectra (**e**) and the intensity (**f**) as the function of gradient Hg^2+^ concentration.

Ion transport in the film can be impaired by the density of the film, preventing the fluorescence quenching of AuNC. It is much more difficult to achieve sufficient quenching efficiency between BSA-AuNC and ions via simple mixing whereas the method of BSA-AuNC printing onto the surface of the CNF membrane can improve this situation. Hence, we considered printing AuNC onto the BSA-AuNC hybrid film to enhance its sensitivity to ions. To this end, we utilized BSA-AuNC as fluorescent ink in a Fuji film inkjet printer (Figure 5a). The printing process was carried out at 50 °C to facilitate water evaporation. “A!”, “A?”, “STOP”, and square patterns were successfully printed, and the printed films presented strong fluorescence under UV irradiation (*λ* = 365 nm) (Figure 5b). The hybrid films printed with BSA-AuNC acted as test strips for testing the response toward various metal ions (10 μM concentration) including Ca^2+^, Co^2+^, Cu^2+^, Cr^3+^, Fe^2+^, Fe^3+^, Mg^2+^, Mn^2+^, Hg^2+^, Ag^+^, and Zn^2+^. Monitoring the changes in fluorescence intensity revealed significant quenching solely for Hg^2+^, with Cu^2+^ displaying minor quenching effects leading to reduced fluorescence intensity (Figure 5d). The interference from most other metal ions was negligible except Cu^2+^, which has a similar electronic structure to Hg^2+^. However, the Hg^2+^ signal was reduced to a larger extent (by more than ¼) compared to Cu^2+^. Hg^2+^ acts as a strong coordination ion, with it able to form stable complexes with ligands on the AuNC surface or in BSA, and the formation of this complex may alter the optical properties of AuNC. There is strong interaction between Hg^2+^ and Au, and this interaction may form a Hg-Au bond, resulting in structural changes in AuNC, thus affecting its fluorescence properties [44]. Under optimized experimental conditions, the sensitivity of the detection system for different concentrations of Hg^2+^ was investigated. As shown in Figure 5e, the fluorescence intensity (at 640 nm) gradually decreased with the increased Hg^2+^ concentration. Such a decline in intensity is supporting evidence for the limit of detection (LOD). The fluorescence intensity decline was caused by Hg^2+^ and can be understood from the standpoint of high-affinity metallophilic Hg^2+^–Au^+^ interaction; Hg^2+^ caused the energy of AuNC excited states to be absorbed or transferred by Hg^2+^ through interaction with AuNC, such as intermolecular resonance energy transfer or electron transfer, thus reducing or preventing the fluorescence emission of AuNC [43]. Moreover, the fluorescence intensity difference was plotted as a function of Hg^2+^ concentration (Figure 5f). The composite film presented high sensitivity, selectivity, and stability. A detection limit of 10 nM was determined, which is the threshold level according to the U.S. EPA standard [45,46]. Compared with previously reported detection methods, our approach is inexpensive and has reasonable sensitivity and selectivity (Table 1):

## 4. Conclusions

To sum up, we have developed a highly sensitive and stable BSA-AuNC/CNF solid-state sensor film for selective heavy metal ion detection (Hg^2+^). The films exhibited cutting-edge performance for the efficient, rapid detection of Hg^2+^. The luminescent (red emission) nanocomposite was shown to be mechanically robust. The printing of BSA-AuNC on the CNF resulted in enhanced ion sensitivity. The fluorescent films, based on CNF, are anticipated to present further potential applications, including in the fields of security features and bioimaging.

## Figures and Tables

**Table 1:** Comparison of our work with the previous colorimetric methods used to detect Hg^2+^.

Samples	Sensing Materials	Methods	Linear Range/Limit of Detection	References
Water	AuNPs stabilized with dithia-diaza ligands	UV–vis absorbance at 680 nm	0–9 μM/35 nM	Woravith et al. [47]
Water	AuNPs	UV–vis absorbance at 652 nm	1–600 nM/0.3 nM	Long et al. [48]
Water	DNA and unmodified AuNPs	UV–vis absorbance (A700 nm/A520 nm)	0–5 mM/0.5 mM	Xu et al. [49]
Water	DNA-Functionalized AuNPs	Melting temperature	0–2 mM/100 nM	Lee et al. [50]
Water	DNA-functioned gold nanoparticles	Fluorescent analysis method AFS	96–6400 nM/40 nM	Wang et al. [51]
Water	BSA-Au clusters	UV–vis absorbance at 630 nm	25 μM/1 nM	Present method

## Data Availability

The original contributions presented in the study are included in the article, further inquiries can be directed to the corresponding authors.
